# Supplementary material for: Rebamipide ameliorates indomethacin-induced small intestinal damage and proton pump inhibitor-induced exacerbation of this damage by modulation of small intestinal microbiota
Source: PLoS One. 2021 Jan 28;16(1):e0245995. doi: 10.1371/journal.pone.0245995 (PMC7842908; doi:10.1371/journal.pone.0245995)
Supplement: S1 Table — (DOCX) [file pone.0245995.s001.docx]

**S1 Table.** The major bacterial composition of small intestine in mice given rebamipide or vehicle at phylum level.

| phylum | control microbiota | rebamipide-modulated microbiota |
| --- | --- | --- |
| *Firmicutes (%)* | 88.48 ± 3.98 | 92.79 ± 2.20 |
| *Bacteroidetes (%)* | 11.02 ± 3.92 | 6.55 ± 2.22 |
| *Proteobacteria (%)* | 0.00 ± 0.00 | 0.13 ± 0.11 |
| *Actinobacteria (%)* | 0.48 ± 0.13 | 0.43 ± 0.11 |
| *Cyanobacteria (%)* | 0.00 ± 0.00 | 0.08 ± 0.06 |

*N* = 8. Values (%) are expressed as mean ± standard error.
