# Supplementary material for: Rebamipide ameliorates indomethacin-induced small intestinal damage and proton pump inhibitor-induced exacerbation of this damage by modulation of small intestinal microbiota
Source: PLoS One. 2021 Jan 28;16(1):e0245995. doi: 10.1371/journal.pone.0245995 (PMC7842908; doi:10.1371/journal.pone.0245995)
Supplement: S2 Table — (DOCX) [file pone.0245995.s002.docx]

**S2 Table.** The major bacterial composition of small intestine in mice given rebamipide or vehicle at genus level.

| genus | control microbiota | rebamipide-modulated microbiota |
| --- | --- | --- |
| *Lactobacillus (%)* | 81.77 ± 4.33 | 84.44 ± 2.18 |
| *Shigella (%)* | 0.00 ± 0.00 | 0.08 ± 0.07 |
| *Dubosiella (%)* | 1.35 ± 0.28 | 0.00 ± 0.00* |
| *Klebsiella (%)* | 0.00 ± 0.00 | 0.03 ± 0.03 |
| *Enterococcus (%)* | 0.00 ± 0.00 | 0.02 ± 0.01* |
| *Blautia (%)* | 0.01 ± 0.01 | 0.04 ± 0.02 |
| *Erysipelatoclostridium (%)* | 0.03 ± 0.01 | 0.05 ± 0.03 |

*N*=8. Values (%) are expressed as mean ± standar error. **p*<0.05 vs control microbiota group.
