# Supplementary material for: Rebamipide ameliorates indomethacin-induced small intestinal damage and proton pump inhibitor-induced exacerbation of this damage by modulation of small intestinal microbiota
Source: PLoS One. 2021 Jan 28;16(1):e0245995. doi: 10.1371/journal.pone.0245995 (PMC7842908; doi:10.1371/journal.pone.0245995)
Supplement: S3 Table — (DOCX) [file pone.0245995.s003.docx]

**S3 Table.** The major bacterial composition of small intestine in mice given rebamipide or vehicle at species level.

| species | control microbiota | rebamipide-modulated microbiota |
| --- | --- | --- |
| *Lactobacillus taiwanensis (%)* | 60.35 ± 5.78 | 39.56 ± 7.89* |
| *Lactobacillus murinus (%)* | 7.34 ± 2.56 | 28.48 ± 9.97* |
| *Lactobacillus reuteri (%)* | 8.88 ± 1.85 | 10.79 ± 2.93 |
| *Shigella dysenteriae (%)* | 0.00 ± 0.00 | 0.07 ± 0.06 |
| *Lactobacillus intestinalis (%)* | 1.49 ± 0.95 | 1.68 ± 0.68 |
| *Dubosiella newyorkensis (%)* | 1.16 ± 0.24 | 0.00 ± 0.00* |
| *Klebsiella oxytoca (%)* | 0.00 ± 0.00 | 0.03 ± 0.03 |
| *Lactobacillus johnsonii (%)* | 0.08 ± 0.02 | 0.04 ± 0.02 |
| *Clostridium cocleatum (%)* | 0.02 ± 0.01 | 0.05 ± 0.03 |
| *Turicibacter sanguinis (%)* | 0.13 ± 0.06 | 0.02 ± 0.01 |
| *Asaccharobacter celatus (%)* | 0.07 ± 0.02 | 0.05 ± 0.03 |
| *Staphylococcus lentus (%)* | 0.02 ± 0.01 | 0.08 ± 0.05 |
| *Schaedlerella arabinosiphila (%)* | 0.03 ± 0.02 | 0.07 ± 0.04 |

*N*=8. Values (%) are expressed as mean ± standard error. **p*<0.05 vs control microbiota group.
