# Supplementary material for: Rebamipide ameliorates indomethacin-induced small intestinal damage and proton pump inhibitor-induced exacerbation of this damage by modulation of small intestinal microbiota
Source: PLoS One. 2021 Jan 28;16(1):e0245995. doi: 10.1371/journal.pone.0245995 (PMC7842908; doi:10.1371/journal.pone.0245995)
Supplement: S4 Table — (DOCX) [file pone.0245995.s004.docx]

**S4 Table.** The major bacterial composition of small intestine in microbiota-transplanted mice administered with omeprazole at phylum level.

| phylum | control microbiota + vehicle | control microbiota + omeprazole | rebamipide-modulated microbiota  + omeprazole |
| --- | --- | --- | --- |
| *Firmicutes (%)* | 79.66 ± 6.61 | 96.12 ± 2.59 | 86.7 ± 4.81 |
| *Bacteroidetes (%)* | 19.60 ± 6.67 | 0.02 ± 0.01** | 7.60 ± 3.66^#^ |
| *Proteobacteria (%)* | 0.60 ± 0.44 | 3.84 ± 2.59 | 5.63 ± 3.71 |
| *Actinobacteria (%)* | 0.12 ± 0.04 | 0.01 ± 0.01* | 0.06 ± 0.02 |
| *Cyanobacteria (%)* | 0.00 ± 0.00 | 0.01 ± 0.01 | 0.00 ± 0.00 |
| *Verrucomicrobia (%)* | 0.01 ± 0.01 | 0.00 ± 0.00 | 0.01 ± 0.01 |

*N* = 6-7. Values are expressed as mean ± SE. **p*<0.05 and ***p* <0.01 vs control microbiota + vehicle group. ^#^*p* <0.05 vs control microbiota + omeprazole group.
