# Supplementary material for: Rebamipide ameliorates indomethacin-induced small intestinal damage and proton pump inhibitor-induced exacerbation of this damage by modulation of small intestinal microbiota
Source: PLoS One. 2021 Jan 28;16(1):e0245995. doi: 10.1371/journal.pone.0245995 (PMC7842908; doi:10.1371/journal.pone.0245995)
Supplement: S5 Table — (DOCX) [file pone.0245995.s005.docx]

**S5 Table.** The major bacterial composition of small intestine in microbiota-transplanted mice administered with omeprazole at genus level.

| genus | control microbiota  + vehicle | control microbiota + omeprazole | rebamipide-modulated microbiota  + omeprazole |
| --- | --- | --- | --- |
| *Lactobacillus (%)* | 66.11 ± 8.33 | 91.49 ± 2.69 | 81.30 ± 5.28 |
| *Robinsoniella (%)* | 8.33 ± 2.31 | 0.09 ± 0.04* | 1.10 ± 0.43 |
| *Shigella (%)* | 0.48 ± 0.39 | 3.10 ± 2.30* | 4.98 ± 3.31 |
| *Klebsiella (%)* | 0.05 ± 0.03 | 0.64 ± 0.41 | 0.51 ± 0.34 |
| *Enterococcus (%)* | 0.19 ± 0.09 | 0.25 ± 0.10 | 0.47 ± 0.10 |
| *Blautia (%)* | 0.03 ± 0.03 | 0.87 ± 0.51 | 0.04 ± 0.02 |
| *Clostridium (%)* | 0.24 ± 0.08 | 0.14 ± 0.08 | 0.03 ± 0.01 |
| *Erysipelatoclostridium (%)* | 0.22 ± 0.10 | 0.01 ± 0.01 | 0.07 ± 0.03 |
| *Terrisporobacter (%)* | 0.23 ± 0.07 | 0.03 ± 0.02* | 0.00 ± 0.00 |

*N*=6-7. Values are expressed as mean ± SE. **p*<0.05 vs control microbiota + vehicle group.
