# Supplementary material for: Rebamipide ameliorates indomethacin-induced small intestinal damage and proton pump inhibitor-induced exacerbation of this damage by modulation of small intestinal microbiota
Source: PLoS One. 2021 Jan 28;16(1):e0245995. doi: 10.1371/journal.pone.0245995 (PMC7842908; doi:10.1371/journal.pone.0245995)
Supplement: S6 Table — (DOCX) [file pone.0245995.s006.docx]

**S6 Table.** The major bacterial composition of small intestine in microbiota-transplanted mice administered with omeprazole at species level.

| species | control microbiota + vehicle | control microbiota + omeprazole | rebamipide-modulated microbiota + omeprazole |
| --- | --- | --- | --- |
| *Lactobacillus taiwanensis (%)* | 38.56 ± 5.92 | 71.37 ± 6.93* | 41.00 ± 4.18^#^ |
| *Lactobacillus murinus (%)* | 8.23 ± 3.81 | 7.70 ± 5.66 | 27.39 ± 2.73^#^ |
| *Lactobacillus reuteri (%)* | 15.49 ± 4.38 | 9.13 ± 2.36 | 7.47 ± 1.42 |
| *Robinsoniella peoriensis (%)* | 7.98 ± 2.24 | 0.08 ± 0.04* | 1.02 ± 0.39 |
| *Shigella dysenteriae (%)* | 0.47 ± 0.38 | 3.00 ± 2.20* | 4.81 ± 3.19 |
| *Lactobacillus intestinalis (%)* | 0.00 ± 0.00 | 0.09 ± 0.07 | 0.00 ± 0.00 |
| *Klebsiella oxytoca (%)* | 0.05 ± 0.02 | 0.64 ± 0.41 | 0.51 ± 0.34 |
| *Blautia hominis (%)* | 0.00 ± 0.00 | 0.79 ± 0.46 | 0.00 ± 0.00 |
| *Enterococcus faecalis (%)* | 0.04 ± 0.02 | 0.17 ± 0.07 | 0.33 ± 0.07* |
| *Lactobacillus johnsonii (%)* | 0.08 ± 0.03 | 0.07 ± 0.03 | 0.07 ± 0.02 |
| *Clostridium cocleatum (%)* | 0.20 ± 0.09 | 0.01 ± 0.01 | 0.02 ± 0.02 |
| *Shigella sonnei (%)* | 0.01 ± 0.01 | 0.10 ± 0.08 | 0.15 ± 0.12 |
| *Enterococcus durans (%)* | 0.15 ± 0.07 | 0.00 ± 0.00 | 0.01 ± 0.01 |
| *Asaccharobacter celatus (%)* | 0.00 ± 0.00 | 0.00 ± 0.00 | 0.01 ± 0.01 |
| *Parabacteroides goldsteinii (%)* | 0.00 ± 0.00 | 0.00 ± 0.00 | 0.14 ± 0.09 |
| *Clostridium butyricum (%)* | 0.08 ± 0.03 | 0.05 ± 0.02 | 0.00 ± 0.00 |

*N*=6-7. Values are expressed as mean ± SE. **p*<0.05 and vs control microbiota + vehicle group. ^#^*p* <0.05 vs control microbiota + omeprazole group.
